# Alternative Processing of Primary microRNA Transcripts by Drosha Generates 5′ End Variation of Mature microRNA

**DOI:** 10.1371/journal.pone.0007566

**Published:** 2009-10-27

**Authors:** Haoquan Wu, Chunting Ye, Danielle Ramirez, N. Manjunath

**Affiliations:** Center of Excellence in Infectious Disease Research, Department of Biomedical Sciences, Paul L. Foster School of Medicine, Texas Tech University Health Sciences Center, El Paso, Texas, United States of America; LMU University of Munich, Germany

## Abstract

**Background:**

It is generally believed that the miRNA processing machinery ensures the generation of a mature miRNA with a fixed sequence, particularly at its 5′ end. However, we and others have recently noted that the ends of a given mature miRNA are not absolutely fixed, but subject to variation. Neither the significance nor the mechanism behind the generation of such miRNA polymorphism is understood. miR-142 is an abundantly expressed miRNA in hematopoietic cells and exhibits a high frequency of 5′ end polymorphism.

**Methodology/Principal Findings:**

Here we show that a shift in the Drosha processing of pri-miRNA generates multiple forms of miR-142s *in vivo* with differing 5′ ends that might target different genes. Sequence analysis of several pre-miRNA ends cloned from T cells reveals that unlike many other pri-miRNAs that are processed into a single pre-miRNA, pri-miR-142 is processed into 3 distinct pre-miR-142s. Dicer processing studies suggest that each of the 3 pre-miR-142s is processed into a distinct double-stranded miRNA, giving rise to 4 mature miRNA variants that might regulate different target gene pools.

**Conclusions/Significance:**

Thus, alternative Drosha processing might be a novel mechanism for diversification of the miRNA target gene pool.

## Introduction

MicroRNAs (miRNAs) are endogenously produced small RNAs that regulate gene expression by translational repression and/or mRNA degradation [1], [2]. miRNAs critically regulate almost all biological processes involving gene expression as evidenced by embryonic lethality in mice deficient in key components of miRNA processing machinery[3], Moreover, alterations in miRNA expression have been implicated in various human disease including caner (reviewed in [4], [5], [6]).

miRNAs are genomically encoded and are transcribed as long primary transcripts (pri-miRNAs). Primary miRNA processing is the first critical step in miRNA biogenesis that crops the mature miRNA out from the long sequence embedded within the pri-miRNA [7]. The pri-miRNA is processed into ∼65 nt hairpin-shaped precursor miRNAs (pre-miRNAs) by a microprocessor complex consisting of the RNase III enzyme Drosha and its cofactor DGCR8 (Pasha in invertebrates) [7], [8], [9], [10], [11], [12]. The molecular basis of miRNA precursor processing has been largely studied by biochemical and mutational analysis *in vitro* [7], [13]. Although an earlier study by Zeng et al suggested that Drosha cuts the pri-miRNA ∼2 helical turns away from the terminal loop [13], a recent study by Han et al suggest that the terminal loop itself is irrelevant and the microprocessor component DGCR8 recognizes both the single-stranded (ss) flanking segment and the double-stranded (ds) RNA stem and then Drosha cuts the pri-miRNA ∼11 nt from the ss-dsRNA junction [7]. They proposed the model based on computational analysis and a series of DGCR8-Drosha processing experiments on pri-miR-16 *in vitro* [7]. However, whether the same rules hold true for miRNAs *in vivo* is not clear. A small number of pre-miRNAs can also be generated as products of mRNA splicing machinery, rather than Drosha processing in Drosophila and C. elegans [14], [15].

The pre-miRNAs are further processed in the cytoplasm by another RNase III enzyme, Dicer that cuts the pre-miRNA ∼22 bp from the end set by Drosha cleavage to generate the double-stranded miRNA duplex [16], [17], [18], [19]. Recruitment of cofactors such as TRBP (Loqs, also known as R3D1, in Drosophila) and PACT increases the efficiency of pre-miRNA processing by Dicer [20], [21], [22], [23], [24], [25]. For most miRNAs, only one strand (the guide strand) of the double-stranded miRNA duplex is loaded into RISC while the other (*) strand is destroyed rapidly [26], [27], [28], [29]. However, in some cases such as miR-142, miR-125, miR-126, miR-342, both strands (5p and 3p) are selected [30]. Schwarz et al proposed that the relative instability of the miRNA duplex termini determines which strand will be loaded into RISC. When both termini of the miRNA have the similar stability, both strands might be selected [26].

MiRNAs are generally imperfectly complementary to the 3′ UTR of the target mRNA, with only 2–7 nt at the 5′ end being perfectly complementary and forms a bulge upon binding to target mRNA [31], [32]. The 5′ 2–7 nt (seed) region defines the target specificity of the miRNA [33], [34], [35] and even a single nucleotide change in the seed region can change the target specificity [36], [37]. Probably because of this, the 5′ end of a given mature miRNA is generally invariant while the 3′ end can exhibit some differences [36], [38], [39], [40], [41].

The miRNA, miR-142 is expressed in all hematopoietic tissues [42], [43] and is one of the most abundantly expressed miRNAs in mouse T cells [41]. Analysis of mature miRNA sequences cloned from mouse T cells suggests that compared to the other abundantly expressed miRNAs, miR-142 is unique in that it exhibits a high frequency of variation at the 5′ end. The 5′ end polymorphism was seen in both miR-142-5p and miR-142-3p. In this report, we identify the cause for the 5′ end changes as a shift in the Drosha processing of pri-miR-142 that generates three distinct isoforms of pre-miR-142s that give rise to four distinct mature miR-142s.

## Results

### Alternative Drosha processing generates multiple pre-miRNAs for miR-142

MiRNA cloning in subsets of T lymphocytes revealed that the sequence of mature miR-142 shows a high degree of polymorphism at the 5′ end compared with the other abundantly expressed miRNAs (Table 1). Notably, in addition to the annotated mature miR-142 sequence in the miRBase, we also cloned some variant forms of miR-142s that appeared to arise as a result of a shift in the processing of the precursors by Drosha/Dicer [41]. For example, some of the clones exhibited 1–3 nucleotides deletion/templated extension at the 5′ end associated with similar number of templated nucleotide extension/deletion at the 3′ end. This kind of variation was seen both in the resting naïve T cells as well as in the more differentiated effector and memory T cells [41]. Moreover, a careful examination of cloned miRNA sequences in the literature revealed that similar variation also occurs in thymic T cells [44]. Because the 5′ end seed region is thought to define the target specificity and even a single nucleotide change in the seed region can change the target specificity [36], [37], the “shift” variants observed in miR-142 might affect target selection.

**Table 1:** Summarized data on the ends of mature miRNAs cloned in vivo.

		dominant variations of 5′ end	total	percent
miR-21	TAGCTTATCAGACTGATGTTGA			
	TAGCTTATCAGACTG…………………	14	14	100%
miR-16	TAGCAGCACGTAAATATTGGCG			
	TAGCAGCACGTAAAT…………………	57	58	98%
miR-150	TCTCCCAACCCTTGTACCAGTG			
	TCTCCCAACCCTTGT…………………	30	30	100%
miR-19b	TGTGCAAATCCATGCAAAACTGA			
	TGTGCAAATCCATGC……………………	6	6	100%
miR-29a	TAGCACCATCTGAAATCGGTTA			
	TAGCACCATCTGAAA…………………	7	8	88%
miR-142-5p	……CATAAAGTAGAAAGCACTACT			
	CCCATAAAGTAGAAA……………………	13	21	62%
	……CATAAAGTAGAAAGC………………	6	21	29%
miR-142-3p	TGTAGTGTTTCCTACTTTATGGA			
	TGTAGTGTTTCCTAC…………………	16	60	27%
	…GTAGTGTTTCCTACT…………………	38	60	63%

Only the first 15 nt of 5′ end of cloned mature miRNA are shown. Sequences exhibiting one or two nt internal mismatches were included in the list. Mature miRNA sequences designated in miRBase are shown in bold.

**Figure 1:**
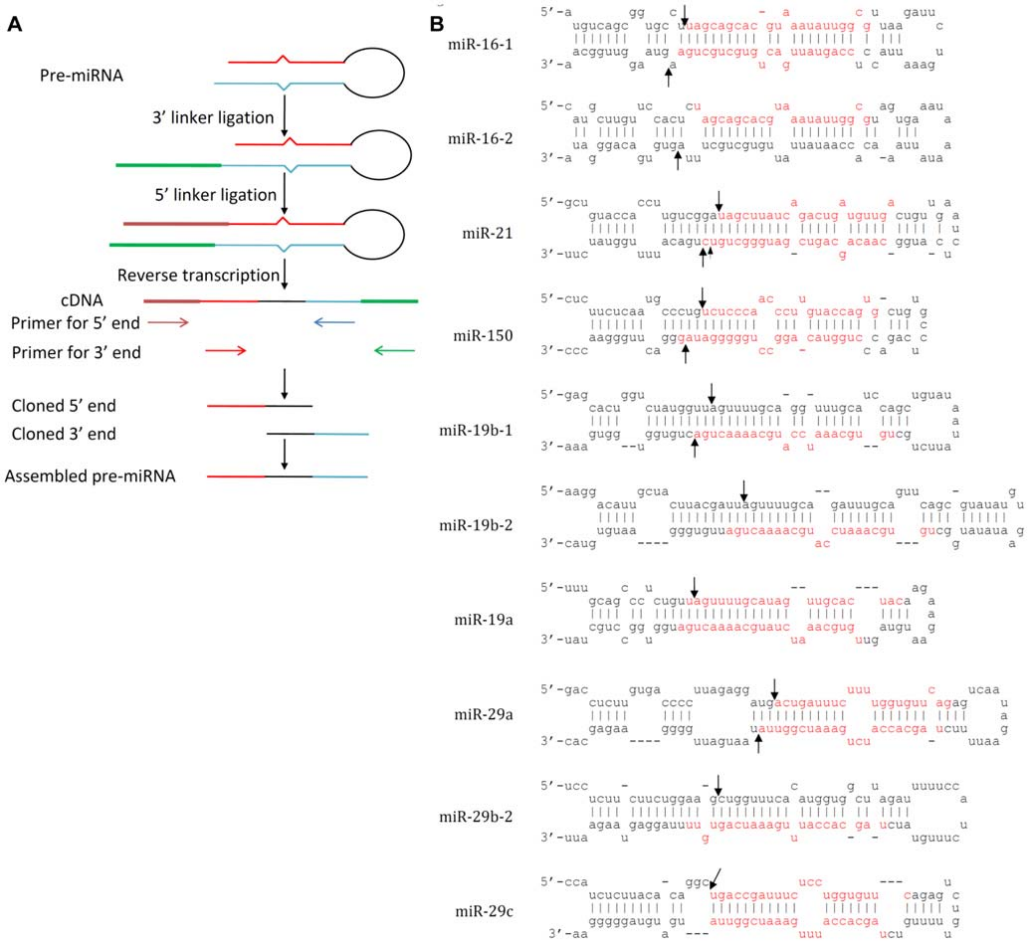
Drosha-DGCR8 process most pri-miRNAs precisely into one pre-miRNA. (A) Schematic of pre-miRNA cloning (see experimental procedures for details). (B) The end cloned and assembled pre-miRNAs are shown. The Drosha cleavage site (deduced from the cloning frequency analysis shown in Table 2) is indicated by arrows. The annotated mature miRNA sequence in miRBase is marked in red.

To understand the mechanism behind the 5′ end variation of mature miRNAs, we attempted to elucidate how pri-miRNAs are processed into pre-miRNAs by Drosha and how pre-miRNAs are further processed by Dicer into mature miRNAs. According to the model proposed by Han et al, DGCR8 acts as a molecular anchor that measures the distance from the dsRNA-ssRNA junction and enables Drosha cleavage to occur ∼11 bp from the stem-ssRNA junction to generate a typical RNase III processed end with 2 nt overhang [7]. Drosha cleavage would therefore set one end of the mature miRNA. Their model was based on computational analysis and a serial of DGCR8-Drosha processing experiments on pri-miR-16 in vitro. However, whether the same rules hold true for miRNAs *in vivo* is not clear. And in a previous study, Basyuk et al were unable to clone let-7 pre-miRNAs with the typical 2 nt overhang structure in vivo and one of the ends of pre-miRNA did not match with the mature miRNA end (implying Drosha cleavage did not always predetermine one end of mature miRNA)[45]. Thus, to better understand how pri-miRNAs are processed into pre-miRNAs *in vivo*, we attempted to clone several pre-miRNAs from murine T cells. Since it is difficult to clone intact pre-miRNAs because of the abundance of other small RNAs of similar length, we designed a method to clone the ends of pre-miRNAs, and then assemble the ends back to pre-miRNAs as illustrated in Figure 1A.

Small RNAs extracted from purified naïve CD8 T cells were used to clone the pre-miRNA ends. We first chose to clone pre-miRNAs for some of the miRNAs that were highly expressed in T cells and where all the clones of mature miRNAs had identical 5′ ends (did not show 5′ end variations). We also included some miRNAs derived from the 3′ arm of miRNA stem loop, such as miR-19 and miR-29. In total, 15 different pre-miRNA ends were cloned (Table 2). Cloning frequency analysis indicates that except miR-21 and miR-29a 3′ ends, all of the ends (both 5′ and 3′) cloned for a given pre-miRNA are identical and the ends of pre-miR-16-1, pre-miR-150, pre-miR-19b-1 and most of pre-miR-21 and pre-miR-29a can be assembled into a prefect RNase III cutting end with 2 nt overhang (Table 2), indicating that Drosha precisely processes one pri-miRNA into one pre-miRNA that has a typical RNase III cutting end with 2 nt overhang *in vivo*(Figure 1B). In the case of pre-miR-21 and pre-miR-29a, although most of 3′ ends could be assembled into a perfect RNaseIII-cutting end with its 5′ ends, small percentage of 3′ ends had variations (Table 2 and Figure 1B) that may represent a modification after Drosha processing. Moreover, for all the miRNAs derived from the 5′ strand of the stem loop, such as miR-21, miR-16 and miR-150, in all clones, the 5′ ends of pre-miRNAs were exactly identical with mature miRNA, suggesting that Drosha has precisely set one end of the mature miRNAs. Thus, in general Drosha processing is precise to generate one pre-miRNA with a typical RNaseIII-cutting 2 nt overhang for a given pri-miRNA, and Drosha processing predetermines one end of mature miRNA.

**Table 2:** Cloned ends of pre-miRNAs.

miR-16-1	template	CUUAGCAGCACGUAAAUAUUGGCGUUAAGAUUCUGAAAUUACCUCCAGUAUUGACUGUGCUGCUGAAG	reads	percent
	5′ ends	……UAGCAGCACGUAAAUAUUGGCGUUAAGAUUCUGAAAUUACCU………………………………………………………………	15	100%
	3′ ends	………………………………………………………GCGUUAAGAUUCUGAAAUUACCUCCAGUAUUGACUGUGCUGCUGAA…	6	100%
miR-16-2	template	UCUAGCAGCACGUAAAUAUUGGCGUAGUGAAAUAAAUAUUAAACACCAAUAUUAUUGUGCUGCUUUAG		
	3′ ends	………………………………………………………GCGUAGUGAAAUAAAUAUUAAACACCAAUAUUAUUGUGCUGCUUUA…	2	100%
miR-21	template	GGAUAGCUUAUCAGACUGAUGUUGACUGUUGAAUCUCAUGGCAACAGCAGUCGAUGGGCUGUCUGACA		
	5′ ends	………UAGCUUAUCAGACUGAUGUUGACUGUUGAAUCUCAUGG………………………………………………………………………	23	100%
	3′ ends	………………………………………………………………ACUGUUGAAUCUCAUGGCAACAGCAGUCGAUGGGCUGUC……………	6	75%
		………………………………………………………………ACUGUUGAAUCUCAUGGCAACAGCAGUCGAUGGGCUGU………………	2	25%
miR-150	template	CCCUGUCUCCCAACCCUUGUACCAGUGCUGUGCCUCAGACCCUGGUACAGGCCUGGGGGAUAGGG		
	5′ ends	……………UCUCCCAACCCUUGUACCAGUGCUGUGCCUCAGACC………………………………………………………………	28	100%
	3′ ends	………………………………………………………………………CUGUGCCUCAGACCCUGGUACAGGCCUGGGGGAUA………	9	100%
miR-19b-1	template	UUAGUUUUGCAGGUUUGCAUCCAGCUGUAUAAUAUUCUGCUGUGCAAAUCCAUGCAAAACUGACUGUG		
	5′ ends	……AGUUUUGCAGGUUUGCAUCCAGCUGUAUAAUAUUCUGCU………………………………………………………………………	19	100%
	3′ ends	…………………………………………………………………UGUAUAAUAUUCUGCUGUGCAAAUCCAUGCAAAACUGA……………	6	100%
miR-19b-2	template	UAGUUUUGCAGAUUUGCAGUUCAGCGUAUAUGUGAAUAUAUGGCUGUGCAAAUCCAUGCAAAACUGAU		
	5′ ends	…AGUUUUGCAGAUUUGCAGUUCAGCGUAUAUGUGAAUAUAUGGCU……………………………………………………………	7	100%
miR19a	template	UUAGUUUUGCAUAGUUGCACUACAAGAAGAAUGUAGUUGUGCAAAUCUAUGCAAAACUGAUGGUGG		
	5′ ends	……AGUUUUGCAUAGUUGCACUACAAGAAGAAUGUAGUU…………………………………………………………………………	10	100%
miR-29a	template	UGACUGAUUUCUUUUGGUGUUCAGAGUCAAUAGAAUUUUCUAGCACCAUCUGAAAUCGGUUAUAAUG		
	5′ ends	……ACUGAUUUCUUUUGGUGUUCAGAGUCAAUAGAAUUUU…………………………………………………………………………	10	100%
	3′ ends	…………………………………………………………AGAGUCAAUAGAAUUUUCUAGCACCAUCUGAAAUCGGUUA……………	29	97%
		…………………………………………………………AGAGUCAAUAGAAUUUUCUAGCACCAUCUGAAAUCGGUUU……………	1	3%
miR-29b-2	template	AGCUGGUUUCACAUGGUGGCUUAGAUUUUUCCAUCUUUGUAUCUAGCACCAUUUGAAAUCAGUGUUUU		
	5′ ends	……CUGGUUUCACAUGGUGGCUUAGAUUUUUCCAUCUUUGUAU……………………………………………………………………	2	100%
miR-29c	template	GGCUGACCGAUUUCUCCUGGUGUUCAGAGUCUGUUUUUGUCUAGCACCAUUUGAAAUCGGUUAUGAUG		
	5′ ends	………UGACCGAUUUCUCCUGGUGUUCAGAGUCUGUUUUUGU…………………………………………………………………………	6	100%

Mature miRNA sequences designated in miRBase are shown in bold.

**Figure 2:**
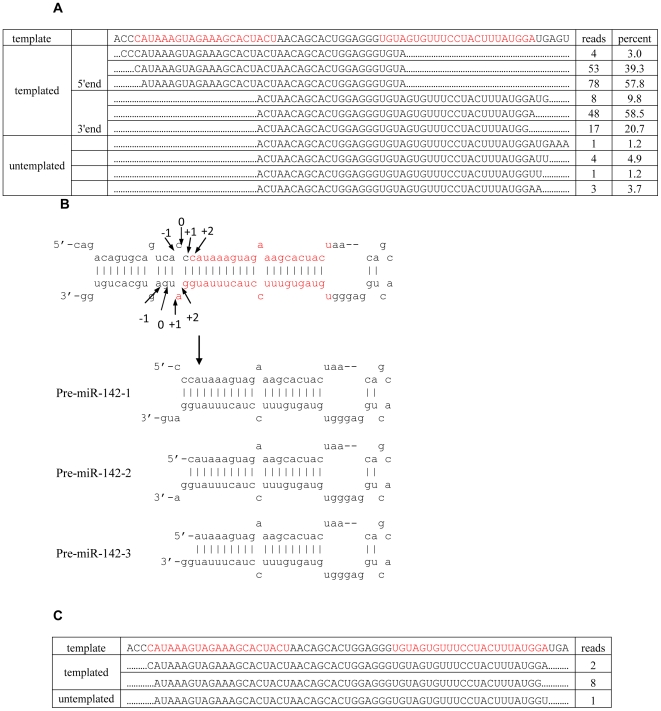
Alternative processing by Drosha-DGCR8 generates three pre-miR-142s from one pri-miR-142. (A). Frequency analysis of the different pre-miR-142 ends cloned. Sequences exhibiting one or two nt internal mismatches were included in the list. (B). Schematic of the generation of 3 pre-miR-142s from one pri-miR142. The alternative Drosha cleavage sites deduced from the cloning data are indicated by arrows. The numeric values represent variations with respect to the predicted cleavage site (0) according to the model proposed by Han et al. The bottom panel shows how alternative processing of pri-miR142 generates 3 pre-miR-142s. (C). Full length pre-miR-142s were pulled down with biotinylated antisense oligos and cloned to visualize the entire sequence. The mature miRNA sequence designated in miRBase is marked in red.

**Figure 3:**
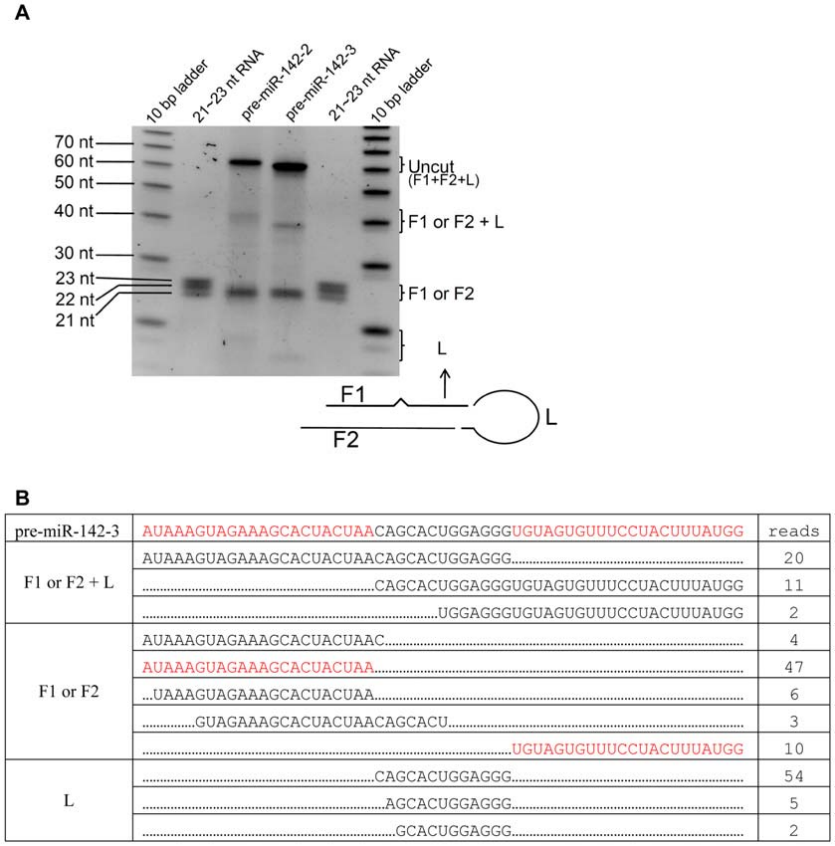
Dicer cuts precisely 22 nt from the end set by Drosha. (A) Synthesized pre-miR-142-2 and pre-miR-142-3 were digested with Dicer, the products separated on 15% urea gel and stained with SybrGold. 10 bp DNA ladder and 21, 22, and 23 nt synthetic RNA oligos were used as molecular weight markers. Note that the marker 21–23 nt RNA oligos lack the 5′ phosphate that Dicer-processed products posses and thus the 22 nt long miR-142s appear to migrate a little faster. The markings F1+F2+L, F1 or F2+L, F1 or F2 and L refers to the undigested, partially digested, fully digested fragments and the terminal loop respectively in lanes 3 and 4. (B). Dicer-processed pre-miR-142-3 fragments were cloned and sequenced. F1 or F2+L represents incompletely digested product (nicked at either 5′ or 3′end), F1 or F2 represents Dicer processed miRNA fragments and L represents the terminal loop. Only the sequences cloned more than twice are listed.

Because mature miR-142 showed 5′ end polymorphism, to test if pri-miR-142 is processed differently, we performed a larger scale of cloning of pre-miR-142 ends using the same RNA as previously used for other pre-miRNAs cloning. Strikingly, both ends of pre-miR-142 showed 3 main variations, and they could be assembled into 3 pre-miR-142s with the typical RNase III cutting end with 2 nt overhang (Figure 2A, B). Thus, it appears that 3 pre-miR-142s are generated from a single pri-miR-142. According to the model proposed by Han et al, the cleavage site for Drosha should be at site 0 as shown in Figure 3B. However, it appears that the exact cleavage site shifted to site −1, +1 and +2 to generate pre-miR-142-1, pre-miR-142-2 and pre-miR-142-3 respectively (Figure 2B). Taken together our results suggest that although Drosha processing is generally precise, it can also generate multiple pre-miRNAs from a single pri-miRNA. Some untemplated addition of nucleotide (A or U) in the 3′ end of pre-miR-142 was also seen at a low frequency, which is reminiscent of the similar untemplated addition in 3′ end of mature miRNAs [36], [38], [39], [40], [41].

To confirm the results obtained from assembling the pre-miRNA ends, we also cloned the pre-miR142 in its entirety. Biotinylated antisense oligos targeting full-length pre-miR-142 were hybridized with small RNAs isolated from T cells and Streptavidin-coupled Dynabeads was used to pull down the pre-miR-142s. Out of the 11 successfully cloned full-length pre-miRNA reads, 8 reads exactly match our predicted pre-miR-142-3 variant and 2 reads matched pre-miR-142-2 (Figure 2C). The 5′ end of the remaining 1 reads matched pre-miR-142-3 but 3′ end has an extra untemplated U, which is consistent with previous data that low frequency untemplated addition of nucleotides (mostly U and A) occurred in the 3′ end of pre-miR-142 (Figure 2A). These results support our finding that multiple pre-miR-142s are generated from one single pri-miR-142.

### Dicer cuts precisely 22nt from the Drosha-processed end

**Figure 4:**
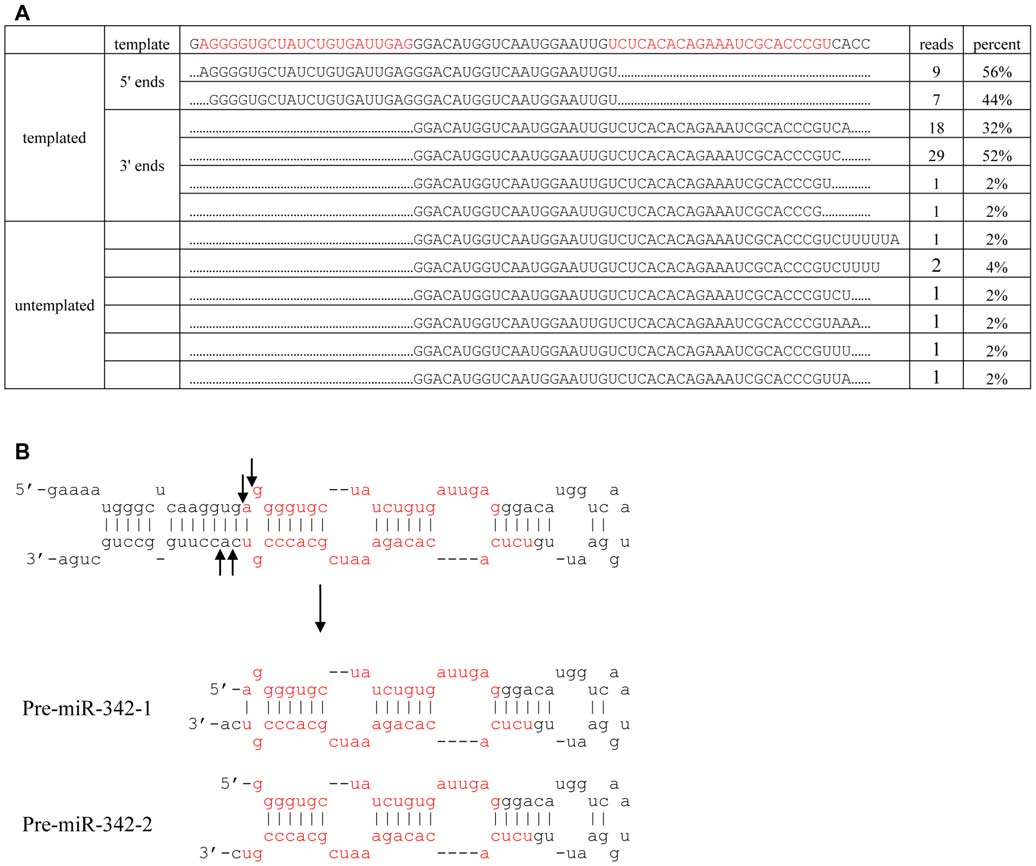
Pri-miR-342 is also alternatively processed by Drosha to generate 2 pre-miRNAs. (A). Cloned pre-miR-342 end frequencies. (B). Schematic showing the processing of Pri-miR-342 into 2 pre-miR-342s. The cleavage sites are indicated with arrows and the mature miRNA sequence designated in the miRBase marked in red.

Dicer has been proposed to measure ∼21 bp from the end set by Drosha cleavage to generate the double-stranded miRNA duplex (ds-miRNA)[16], [17], [18], [19]. So we hypothesized that the 3 pre-miR-142s seen in T cells should be processed into 3 double-stranded miRNAs (ds-miRNAs) by Dicer. To verify the hypothesis, we performed *in vitro* Dicer cleavage assay. Pre-miR-142-2 and pre-miR-142-3 oligonucleotides were synthesized and digested with Dicer. As shown in Figure 4A, Dicer digested both pre-miR-142-2 and pre-miR-142-3 predominately into a 22 nt product. The intermediate products of ∼40 bp seen in Figure 3A (F1+L or F2+L) appears to be due to the nonsimultaneous cleavage of 5′ and 3′ strand of the miRNAs by one RIII domain of Dicer (Dicer has two tandem RIII domains that cut at 5′ strand and 3′ strand separately [46]). Also we could visualize the band of terminal loop (L) that is around 15 nt. The intermediate product (F1+L or F2+L) and terminal loop (L) of pre -miR-142-2 are about 2 nt longer than that of pre-miR-142-3, indicating that Dicer measures ∼22 nt from the end set by Drosha processing and makes a 1 nt shift accordingly (Figure 3A).

Although Dicer has been under intensive investigation for long time and it has been proposed that Dicer cleaves pre-miRNA ∼21 nt from the end set by Drosha cleavage [16], [19], [47], almost no study has directly verified the exact Dicer cleavage site of pre-miRNA. Thus, to confirm the exact Dicer cleavage site, we cloned the cleavage products of Dicer-processed pre-miR-142-3. The sequences of the different Dicer cleavage products indicate that Dicer cuts precisely at 22 nt from the site set by Drosha to generate a typical RNase III end with 2 nt overhang (Figure 3B).

Interestingly, we did not see the 3′ end polymorphism during *in vitro* Dicer processing (deletion like modification and untemplated addition) that are commonly observed in the small RNA cloning data for *in vivo* mature miRNAs [36], [38], [39], [40], [41]. It has been long suspected that the end polymorphism might be an artifact of cloning procedure. Our data show that small RNA cloning procedure in itself did not generate that kind of variation, suggesting that the 3′ end polymorphism found in mature miRNA cloning is more likely not an artifact.

### Another example of Drosha processing shift

Because thermodynamic parameters dictate miRNA strand selection by RISC, the shift in pri-miRNA processing may also aid in the selection of both strands: for example, the structure of double-stranded miR-142-1 (ds-miR-142-1) favors selection of miR-142-5p-V1 while ds-miR-142-3 favors selection of miR-142-3p-V1. From our mature mi-RNA cloning data, it does appear that indeed both strands are selected [41]. However, they are actually derived from 2 different double-stranded miRNA (ds-miRNA) duplexes, and the 2 selected strands do not make a typical ds-miRNA having 2 nt overhang. Thus, we hypothesized that some other miRNAs where both 5′ and 3′ strands get selected into RISC and the 2 mature miRNA strands do not form a typical ds-miRNA with 2 nt overhang might also exhibit Drosha processing shift. miR-342 has both its strand selected and the two mature miRNAs (miR-342-5p and miR-342-3p) appear not to make a perfect 2 nt overhang Drosha/Dicer processed product. To test if this might be a result of a processing shift, we cloned the ends of pre-miR-342 from the same RNAs used for other pre-miRNAs cloning. The results show both ends of miR-342 have two dominant variations respectively, and can be assembled into 2 perfect pre-miR-342s (Figure 4), suggesting that Drosha processing shift occurs in miR-342 and generates two pre-miR-342s from one pri-miR-342. Also consistent with what was observed with pre-miR-142, the 3′ ends of pre-miR-342 showed two dominant variations as well as some untemplated addition of nucleotide (A or U) at a low frequency.

## Discussion

**Figure 5:**
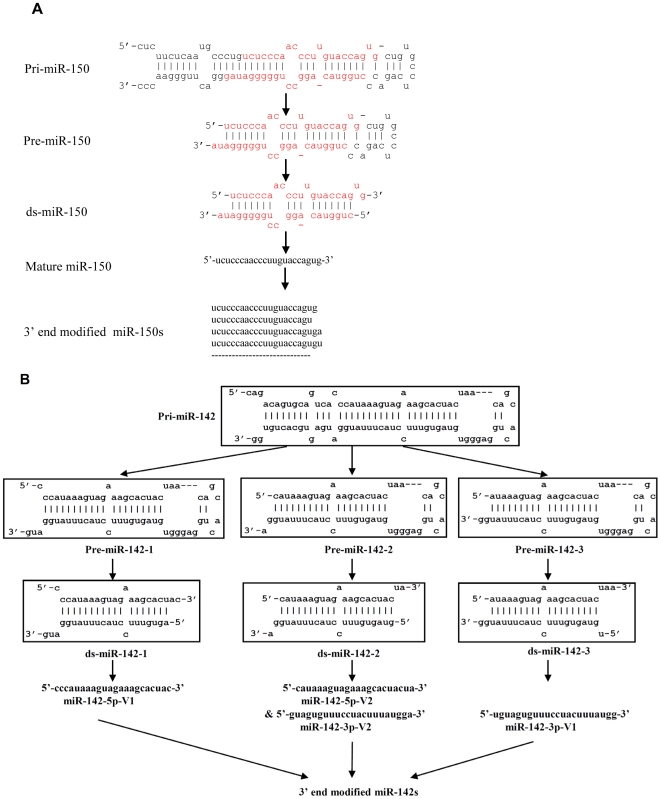
A model for the generation of mature miRNA end polymorphism. (A). The biogenesis of class I miRNA (represented by miR-150): here the Drosha cleavage is precise and generates only one pre-miRNA from the pri-miRNA and hence the end is fixed and does not show polymorphism. Since the end of pre-miRNA is homogenous and Dicer processing is also precise, the 3′ end polymorphism seen in the mature miRNA is more likely generated subsequent to Drosha/Dicer processing. (B). The biogenesis of class II miRNAs. Here, because alternative processing of pri-miRNA by Drosha generates multiple pre-miRNAs that are each processed precisely by Dicer, the 5′ end of mature miRNA exhibits polymorphism. The 3′ end polymorphism arises after Drosha/Dicer processing as explained in (A).

It is generally believed that the miRNA processing machinery ensures the generation of a mature miRNA with a fixed sequence, particularly at its 5′ end. However, it is increasingly being recognized that the ends of a given mature microRNA is not absolutely fixed, but is subject to variation in C elegans [40], mammalian cells [41] as well as in Drosophila [36]. Neither the significance nor the mechanism behind the generation of such miRNA polymorphism is understood. Based on our *in vivo* pre-miRNA cloning results, here we propose a model for the generation of end polymorphism as illustrated in Figure 5. We propose that miRNAs can be divided into 2 categories according to how pri-miRNAs are processed into pre-miRNAs. For most miRNA (10 out of 12 studied here, we designate them as class I miRNAs), Drosha processing is precise and generates only one pre-miRNA from one pri-miRNA while for a few other miRNAs (such as miR-142 and miR-342, we designate them as class II miRNAs), more than one dominant pre-miRNA is generated from one pri-miRNA because of a shift in the processing by Drosha. Dicer processing of these alternatively processed pre-miRNAs then generates mature miRNAs showing variations at the 5′ end that will diversify the miRNA target gene pool.

It has recently been proposed that imprecision in the Drosha/Dicer processing may be the reason for mature miRNA end polymorphism [36], [40]. However our results suggest that Drosha processing *in vivo* appears to be very precise for class I miRNAs. Actually all of the 5′ ends and most 3′ ends of pre-miRNAs we cloned *in vivo* support our argument (Table 2), and we believe those low frequency exceptional reads observed in pre-miR-21 and pre-miR-29a 3′ ends are modified after Drosha processing. *In vitro* Dicer processing of pre-miRNAs also show that Dicer processing is generally precise and it cuts ∼22 nt from the Drosha-set end to generate the double-stranded miRNA (ds-miRNA). Therefore, a single species of ds-miRNA (with invariant 5′ and 3′ ends) should be generated after Drosha/Dicer processing of this class of miRNAs. However, mature miRNA cloning data even for class I miRNAs shows considerable polymorphism at the 3′ end [36], [38], [40], [41]. Thus, the 3′ end polymorphism is more likely generated after Drosha/Dicer processing as illustrated in Figure 5A. The pre-miRNA cloning data for miRNAs generated from the 3′ arm of the stem loop also provide another powerful evidence to support our model. Mature miR-19b and miR-29a are derived from 3′ arm of the stem loop, so the pre-miRNA 3′ end is exactly the 3′ end of mature miRNA. Cloning frequency analysis shows that pre-miR-19b and pre-miR-29a have almost identical 3′ ends (only 1 out of 36 is not identical. Table 2) while the 3′ ends of mature miRNA are highly varied [41], [44]. The 5′ ends of mature miR-19b and miR-29a are processed by Dicer and they are identical. Thus, the 3′ end polymorphism of miR-19b and miR-29a is more likely generated after Drosha/Dicer processing, not due to imprecision of Drosha/Dicer processing.

For the class II miRNAs, Drosha wavers to generate multiple pre-miRNAs from a single pri-miRNA. The alternative processing did not occur randomly, but only in a few particular miRNAs, such as miR-142 and in a well-controlled manner. This shift in Drosha processing generates 3 pre-miR-142s that are processed into 3 ds-miRNAs by Dicer (Figure 5B). Schwarz et al has proposed that the relative instability of the ds-miRNA termini determines which strand will be loaded into RISC [26]. According to this model, since the termini of miR-142-2 have almost the same stability, both strands should be selected to generate miR-142-2-5p and miR-142-2-3p. On the other hand, miR-142-1 should generate a miR-142-1-5p while miR-142-3 should generate a miR-142-3-3p (Figure 5B). This prediction exactly matches what we observed for *in vivo* mature miR-142 in our previous study—4 dominant 5′ end variation (Table 1) and many more 3′ end variations [41], [44], probably generated subsequent to Drosha/Dicer processing. Thus, it appears that the 5′ end polymorphism confined to a few miRNAs is a result of Drosha processing shift while the more common 3′end variations observed in most miRNAs is due to modifications introduced after the Drosha/Dicer processing. This model may explain why 5′ end polymorphism of mature miRNA only occur in a few particular miRNAs and why 3′ end is always much more heterogeneous than 5′ end.

Han et al has proposed that Drosha processing predetermines one end of mature miRNA [7]. Their conclusion is mainly based on the miRNAs derived from 5′ arm of the pre-miRNA stem loop, such as miR-16 and miR-30a. In these miRNAs, Drosha cleavage generates the 5′ end of the mature miRNA and the 5′ ends are usually invariant in the mature miRNA, so it is easy to deduce that Drosha processing determines one end of the mature miRNA. However, it is difficult for the miRNAs derived from 3′ arm of pre-miRNA stem loop since Drosha processing generates the 3′ end of the mature miRNA. As mentioned before, the 3′ end of mature miRNA is highly varied, so it will be difficult to deduce from the mature miRNA sequence where Drosha cuts exactly. Our data suggest that Drosha processing set precisely one end of mature miRNA, no matter the miRNA is derived from 5′ or 3′ arm (Figure 1B). Dicer just measures from this end and generates the other end of mature miRNA (Figure 3). Thus, Drosha processing actually has predetermined both ends of mature miRNA and therefore, appears to be the most critical step of miRNA biogenesis.

### An alternatemechanism for the selection of both strands of some miRNAs

**Figure 6:**
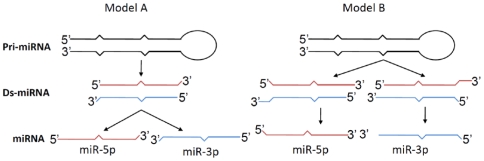
Different models for the selection of both strands of a miRNA. Two mechanisms might operate for both strand selection: Model A as proposed by Schwarz et al [26], where the termini of ds-miRNA duplex have similar thermodynamic stability and both strands in the ds-miRNA are selected. Model B might operate in cases where the two strands are derived from different ds-miRNAs, one with thermodynamic feature favoring selection of 5p and the other favoring selection of 3p. This mechanism might be responsible when the two selected strands do not form a perfect ds-miRNA with 2 nt overhangs.

For most miRNAs, only one guide strand will be loaded into RISC while the other strand is destroyed rapidly [26], [27], [28], [29]. However in some cases, such as miR-142, miR-125, miR-126, miR-342 etc, both strands are selected [30]. The biogenesis of miR-142 provides evidence how both strands are selected. As illustrated in Figure 6, there are 2 models that can explain the phenomenon. Model A has been proposed by Schwarz et al: when both termini of the ds-miRNA have the similar stability, both strands might be selected (Figure 6A) [26]. That is clearly the case for ds-miR-142-2—both its termini have the similar stability, and both strands (miR-142-5p-V2 and miR-142-3p-V2) are selected (Figure 5B). This model can fit well with miRNAs for which both strands are selected and the two strands make a perfect ds-miRNA with similar stability at both ends. However, it does not fit for miRNAs for which the two selected strands do not make a perfect ds-miRNA with similar stability on both termini. Here we propose an alternative model to explain the latter situation. Model B is proposed based on our data that there are multiple ds-miRNAs generated from one pri-miRNA and they have differing stability at the termini (Figure 6B). ds-miR-142-1 and ds-miR-142-3 have opposite termini stability and generate miR-142-5p-V1 and miR-142-3p-V1 respectively (Figure 5B). miR-142-5p-V1 and miR-142-3p-V1 do not make a perfect ds-miRNA with a 2 nt overhang. Although it appears that both strands could be selected from either of them, they are actually derived from two different ds-miRNAs derived from one pri-miRNA due to Drosha processing shift. Thus, the hallmark for model B is that the 2 strands selected by RISC do not make a perfect ds-miRNA with 2 nt overhang. In fact, we identified that miR-342 might be another example of Drosha processing shift because although its both strands are selected, the two strands do not make a typical ds-miRNA that has 2 nt overhang in both termini. In summary, both model A and model B appear to operate and based on whether two strands make a typical ds-miRNA, it will not be difficult to predict which model is responsible.

### Alternative Drosha processing might be a conserved phenomenon

Although not alluded to by the authors, a careful analysis of the miRNA sequences in the literature also reveals other examples of 5′ end variations in the mature miRNA. Examples include mmu-miR-151 in mouse [48], has-miR-368 in human [30]. Also some of miRNAs in C. elegans, such as miR-49, miR-228, miR-229*, miR-238, miR-240, miR-246, and miR-788 have high frequency of 5′ end variation [40]. It is possible that many more miRNAs with 5′ shifts will be found in the future. In fact, Seitz et al have also recently reported on the existence of 5′ end variation in Drosophila mature miRNAs [36]. Just like in mice, most miRNAs in Drosophila have identical 5′ end. However, some miRNAs, such as miR-100, miR-283, miR-33*, miR-14*, have variable 5′ end. So it appears that the 5′ end polymorphism in some particular miRNAs is a conserved phenomenon in C. elegans, Drosophila, mouse and human. These 5′ end polymorphisms are likely also the consequence of a Drosha processing shift. Taken together, Drosha processing shifts may be a not uncommon event that has been largely ignored in the literature so far.

### Diversification of miRNA target pool by alternative processing of pri-miRNA

The 5′ 2–7 nt of a microRNA determines the target specificity and even a single nucleotide change can change target selection [33], [34], [35]. Thus, it is generally believed that mature miRNA sequence is fixed, particularly at its 5′end. Our results show that this theory although generally true, is not invariable. Our results suggest that in particular micro-RNAs such as miR-142, Drosha can shift the processing sites of pri-miRNA precursor to generate alternative pre-miRNA variants that can then be independently processed by Dicer to generate mature miRNAs that might have altered target specificity. The seed-matched target-binding sites of the different miR-142 variants appear to be highly evolutionarily conserved. According to TargetScan, the seed sequence (ccauaaa) of miR-142-5p-V1 (cccauaaaguagaaagcacuac) matches 269 conserved binding sites in 263 target genes while the seed sequence (auaaagu) of miR-142-5p-V2 (cauaaaguagaaagcacuacua) matches 440 conserved binding sites in 422 target genes; the seed sequence (guagugu) of miR-142-3p-V1 (uguaguguuuccuacuuuaugg) matches 227 conserved binding sites in 212 target genes while the seed sequence (uaguguu) of miR-142-3p-V2 (guaguguuuccuacuuuaugga) matches 399 conserved binding site in 369 target genes. Some of the targets are overlapped with each other while most are separate, indicating that these distinct miR-142 variations might regulate different target gene pools. It is also noteworthy that miR-142 is the most highly expressed miRNA in naïve T cells, the concentration of each of the individual miR-142 variants is much higher than most miRNAs expressed in naïve T cells. Moreover the predominant miR-142 isoform differs in sequence than the annotated sequence in the miR-base. All these evidences suggest that alternative Drosha processing generated multiple miR-142 variants might be physiologically significant to diversify the target gene pools regulated by miR-142. Interestingly, the proportion of different variants appears to change with T cell development. For example, in the thymic double negative T cells, miR-142-3p-V2 was the predominant variant, while in mature naïve CD8 T cells, the dominant variant was miR-142-3p-V1 [41], [44]. Thus, the processing shift might be a mechanism to fine tune gene expression and the change of dominant variants in different cell stages might add more flexibility to regulate target genes during T cell differentiation.

How exactly the shift in Drosha processing occurs remains to be determined. However, factors other than the microprocessor components themselves are known to affect processing. For e.g., post-transcriptional modification of pri-miRNAs has been reported. Pri-miR-142 can be modified by A to I editing by ADAR and this modification can inhibit Drosha processing [37].Thus, it is possible that site-specific modification by ADAR or other unidentified proteins may facilitate alternative processing. Recently, multiple reports have suggested that the processing of pri-miRNA into pre-miRNA is an important regulatory step for miRNA biogenesis. The DEAD-box RNA helicase subunits p68 and p72, TGF-b and BMP-specific-SMAD signal transducers as well as the tumor suppressor p53 have all been reported to associate with the Drosha complex and influence pri-miRNA processing [49], [50], [51]. Guil et al also reported that the multifunctional RNA-binding protein hnRNP binds pri-miR-18a and regulate pre-miR-18a level [52]. Viswanathan et al reported that Lin28 selectively blocks pri-let-7 being processed into pre-let-7[53]. Thus it appears that many aspects of pri-miRNA processing are yet to be fully understood.

## Materials and Methods

### Pre-miRNA ends cloning

RNA was isolated from purified mouse naïve CD8 T cells as described earlier [41] using Qiagen miRNeasy kit according to the manufacturer's instruction. The cloning method was modified from previously described protocol [44]. Briefly, small RNAs were ligated to 3′ adenylated linker (5′-rAppCTGGTATCTGTGTATGGddC-3′) in a buffer without ATP, followed by 5′ linker (5′-ACCACAGAGAAACCGrCrArG-3′). The ligated short RNAs were then separated on 15% polyacrylamide urea gels and fragments between 70 to 200 nt was cut out and purified. The ligated RNAs were reverse transcribed and PCR amplified using the oligo primer sequences listed in Table S1. The amplified DNAs were separated on 4% agarose gel (fragment 10–500 bp agarose, US Biological) and fragments around 100 bp was cut out and purified. The DNAs were amplified again, concatamerized and cloned into T vector and sequenced at Functional Bioscience, Inc.

### In vitro Dicer cleavage assay

RNA oligonucleotides of two pre-miRNAs, pre-miR-142-2 and pre-miR-142-3 were synthesized with a phosphorylated 5′ end (Dharmacon). For *in vitro* Dicer cleavage assays, recombinant Dicer enzyme was used (Genlantis/Gene Therapy Systems, San Diego, CA). In the cleavage reaction, 200 pmol pre-miRNA was incubated with 1 unit Dicer at 37°C for 4 hr. A portion of the reaction mixture (1/10) was denatured and loaded into a 15% TBE-Urea gels (Invitrogen). After electrophoresis, the gel was stained with SYBR Gold (Invitrogen). The remaining Dicer cleavage reaction mixture was extracted with phenol/chloroform, and precipitated with ethanol. The small RNAs were cloned according to the protocol described previously [44]. The only difference here was that after amplification, the different sized products were separated on a 4% agarose gel (US Biological) and cloned separately.

### Cloning of pre-miR-142 in its entirety

Small RNA fraction extracted from naïve T cell was separated by 15% TBE-urea gel. The pre-miRNA fraction (roughly 50–70 nt) was excised and purified. 5′ and 3′ linker was added to the purified pre-miRNA portion RNAs. The RNA was incubated with 5′-/5BioTEG/AGTAGG AAACACTACACCCTCCAGTGCTGTTAGTAGTGCTTTCTAC-3′ at 50°C for 1 hours, followed by incubation with Dynabeads® M-280 Streptavidin for 1 hour at room temperature. After washing, the RNA was reverse-transcribed and cloned as described earlier [41].

## Supporting Information

Table S1 Primers used for cloning the pre-miRNA ends.
(0.05 MB DOC)
