# Supplementary material for: Alternative Processing of Primary microRNA Transcripts by Drosha Generates 5′ End Variation of Mature microRNA
Source: PLoS One. 2009 Oct 27;4(10):e7566. doi: 10.1371/journal.pone.0007566 (PMC2762519; doi:10.1371/journal.pone.0007566)
Supplement: Table S1 — Primers used for cloning the pre-miRNA ends. (0.05 MB DOC) [file pone.0007566.s001.doc]

**Table S1**

| Pre-miR-16-1 5’ end | forward | GAGCCAACAGGCACCACAGAGAAACCGCAG |
| --- | --- | --- |
|  | reverse | GACTAGCTTGGTGCCGCAGCACAGTCAATACTGG |
| Pre-miR-16-1 3’ end | forward | GAGCCAACAGGCACCAGCAGCACGTAAATATTG |
|  | reverse | GACTAGCTTGGTGCCATACACAGATACCAG |
| Pre-miR-21  5’ end | forward | GAGCCAACAGGCACCACAGAGAAACCGCAG |
|  | reverse | [GACTAGCTTGGTGCC](https://www.idtdna.com/OrderStatus/SpecSheet.aspx?OrderNum=4802835&MfgID=38295436&MfgLocID=1&SearchDays=&SearchNum=&SearchPO=&SearchRef=&ProdID=1213)GACAGCCCATCGACTGCTGTTG |
| Pre-miR-21  3’ end | forward | [GAGCCAACAGGCACC](https://www.idtdna.com/OrderStatus/SpecSheet.aspx?OrderNum=4802835&MfgID=38295434&MfgLocID=1&SearchDays=&SearchNum=&SearchPO=&SearchRef=&ProdID=1213)TAGCTTATCAGACTGATGTTG |
|  | reverse | GACTAGCTTGGTGCCATACACAGATACCAG |
| Pre-miR-150 5’ end | forward | GAGCCAACAGGCACCACAGAGAAACCGCAG |
|  | reverse | [GACTAGCTTGGTGCC](https://www.idtdna.com/OrderStatus/SpecSheet.aspx?OrderNum=4802835&MfgID=38295436&MfgLocID=1&SearchDays=&SearchNum=&SearchPO=&SearchRef=&ProdID=1213)CTATCCCCCAGGCCTGTACCAG |
| Pre-miR-150 3’ end | forward | [GAGCCAACAGGCACC](https://www.idtdna.com/OrderStatus/SpecSheet.aspx?OrderNum=4802835&MfgID=38295434&MfgLocID=1&SearchDays=&SearchNum=&SearchPO=&SearchRef=&ProdID=1213)TCTCCCAACCCTTGTACCAGTG |
|  | reverse | GACTAGCTTGGTGCCATACACAGATACCAG |
| Pre-miR-29a 5’ end | forward | GAGCCAACAGGCACCACAGAGAAACCGCAG |
|  | reverse | GACTAGCTTGGTGCCAACCGATTTCAGATGGTGCTAG |
| Pre-miR-29a 3’ end | forward | GAGCCAACAGGCACCTGATTTCTTTTGGTGTTCAG |
|  | reverse | GACTAGCTTGGTGCCATACACAGATACCAG |
| Pre-miR-19b-1 5’ end | forward | GAGCCAACAGGCACCACAGAGAAACCGCAG |
|  | reverse | [GACTAGCTTGGTGCC](https://www.idtdna.com/OrderStatus/SpecSheet.aspx?OrderNum=4802835&MfgID=38295436&MfgLocID=1&SearchDays=&SearchNum=&SearchPO=&SearchRef=&ProdID=1213)TCAGTTTTGCATGGATTTGCAC |
| Pre-miR-19b-1 3’ end | forward | [GAGCCAACAGGCACC](https://www.idtdna.com/OrderStatus/SpecSheet.aspx?OrderNum=4802835&MfgID=38295434&MfgLocID=1&SearchDays=&SearchNum=&SearchPO=&SearchRef=&ProdID=1213)AGTTTTGCAGGTTTGCATCCAGC |
|  | reverse | GACTAGCTTGGTGCCATACACAGATACCAG |
| Pre-miR-142 5’ end | forward | GAGCCAACAGGCACCACAGAGAAACCGCAG |
|  | reverse | GACTAGCTTGGTGCCATAAAGTAGGAAACAC |
| Pre-miR-142 3’ end | forward | GAGCCAACAGGCACCATAAAGTAGAAAGCACT |
|  | reverse | GACTAGCTTGGTGCCATACACAGATACCAG |
| Pre-miR-342 5’ end | forward | GAGCCAACAGGCACCACAGAGAAACCGCAG |
|  | reverse | [GACTAGCTTGGTGCC](https://www.idtdna.com/OrderStatus/SpecSheet.aspx?OrderNum=4802835&MfgID=38295436&MfgLocID=1&SearchDays=&SearchNum=&SearchPO=&SearchRef=&ProdID=1213)GGGTGCGATTTCTGTGTGAG |
| Pre-miR-342 3’ end | forward | [GAGCCAACAGGCACC](https://www.idtdna.com/OrderStatus/SpecSheet.aspx?OrderNum=4802835&MfgID=38295434&MfgLocID=1&SearchDays=&SearchNum=&SearchPO=&SearchRef=&ProdID=1213)AGGGGTGCTATCTGTGATTGAG |
|  | reverse | GACTAGCTTGGTGCCATACACAGATACCAG |
